# Complete Genome Sequence of the Highly Virulent *Aeromonas schubertii* Strain WL1483, Isolated from Diseased Snakehead Fish (*Channa argus*) in China

**DOI:** 10.1128/genomeA.01567-15

**Published:** 2016-01-21

**Authors:** Lihui Liu, Ningqiu Li, Defeng Zhang, Xiaozhe Fu, Cunbin Shi, Qiang Lin, Guijie Hao

**Affiliations:** a Key Laboratory of Fishery Drug Development, Ministry of Agriculture Key Laboratory of Aquatic Animal Immune Technology, Pearl River Fishery Research Institute, Chinese Academy of Fishery Sciences, Guangzhou, Guangdong, China; b Agriculture Ministry Key Laboratory of Healthy Freshwater Aquaculture, Key Laboratory of Fish Health and Nutrition of Zhejiang Province, Zhejiang Institute of Freshwater Fisheries, Huzhou, Zhejiang, China; c Freshwater Aquaculture Collaborative Innovation Center of Hubei Province, Wuhan, Hubei, China

## Abstract

We sequenced the complete genome of the highly virulent *Aeromonas schubertii* strain WL1483, which was isolated from diseased snakehead fish (*Channa argus*) in China. The full genome sequence of *A. schubertii* WL1483 is 4,400,034 bp, which encodes 4,376 proteins and contains 195 predicted RNA genes.

## GENOME ANNOUNCEMENT

*Aeromonas schubertii* is a Gram-negative, short rod-shaped bacterium, commonly isolated from abscesses, wounds, skin, and pleural fluid (1–3). It can cause septicemia with necrotizing fasciitis and posttraumatic soft tissue infection, especially with trauma associated with freshwater environment or marine animals (4). *A. schubertii* was first confirmed as the etiological agent of the epizootic in cultured snakehead fish and is becoming a major economic problem in the snakehead farming industry (5, 6). The strain *A. schubertii* WL1483 was a representative isolate collected from a 2014 disease outbreak among snakeheads in earthen ponds in Foshan, Guangdong Province, China. Virulence studies revealed that *A. schubertii* WL1483 was highly virulent to snakehead fish (*Channa argus*). The pathogenesis of *A. schubertii* is not understood as yet, and fewer sequences of *A. schubertii* are available. Therefore, the complete genome sequence of *A. schubertii* WL1483 was determined in this study.

Genomic DNA was extracted from the isolate *A. schubertii* strain WL1483, according to the protocol of the Bacterial DNA kit (OMEGA, USA). The DNA was sequenced using the PacBio RSII and Illumina HiSeq platforms. The hierarchical genome assembly process from the Pacific Biosciences single-molecule real-time analysis toolkit (7) was used to obtain a *de novo* assembly. The PacBio RSII reads were aligned to the trimmed assembly using BLAST to correct the structure error. The original reads of HiSeq were used to fill outer gaps and correct the assembly results. The results showed an assembly with a single contig and a sequence length of 4,400,034 bp, with an average coverage depth of 587.5×. The genome sequence has a mean G+C content of 61.49%.

The assembled sequence was annotated using the GeneMarkS annotation system for prokaryotic genomes (8). tRNA genes were predicted with tRNAscan-SE (9), rRNA genes were predicted with rRNAmmer (10), and sRNAs were predicted by BLAST against the Rfam database (11). A total of 4,376 protein-coding sequences were identified, together with 107 tRNA genes, 81 rRNA genes, and 7 sRNA genes. Protein annotation using the VFDB database (http://www.mgc.ac.cn/VFs) of virulent factors for bacterial pathogens detected 365 putative virulence factors, and 308 secretory proteins were detected on the genome assembly by SignalP (12).

### Nucleotide sequence accession numbers.

The sequence and annotation of *A. schubertii* strain WL1483 has been deposited at GenBank under the accession number CP013067, BioSample number SAMN04110141, and BioProject number PRJNA297116.
